# Temsavir Treatment of HIV-1-Infected Cells Decreases Envelope Glycoprotein Recognition by Broadly Neutralizing Antibodies

**DOI:** 10.1128/mbio.00577-22

**Published:** 2022-04-27

**Authors:** Marianne Boutin, Dani Vézina, Shilei Ding, Jérémie Prévost, Annemarie Laumaea, Lorie Marchitto, Sai Priya Anand, Halima Medjahed, Gabrielle Gendron-Lepage, Catherine Bourassa, Guillaume Goyette, Andrew Clark, Jonathan Richard, Andrés Finzi

**Affiliations:** a Centre de Recherche du CHUM (CRCHUM), Montreal, Quebec, Canada; b Département de Microbiologie, Infectiologie et Immunologie, Université de Montréal, Montreal, Quebec, Canada; c Department of Microbiology and Immunology, McGill University, Montreal, Quebec, Canada; d ViiV Healthcare, Global Medical Affairs, Middlesex, United Kingdom; University of Pittsburgh School of Medicine

**Keywords:** HIV-1, Env glycoprotein, entry inhibitors, attachment inhibitors, fostemsavir, BMS-663068, temsavir, BMS-626529, glycosylation, proteolytic cleavage, antibody-dependent cellular cytotoxicity, ADCC, Env cleavage, broadly neutralizing antibodies, bNAbs

## Abstract

The heavily glycosylated HIV-1 envelope glycoprotein (Env) is the sole viral antigen present at the surface of virions and infected cells, representing the main target for antibody responses. The FDA-approved small molecule temsavir acts as an HIV-1 attachment inhibitor by preventing Env-CD4 interaction. This molecule also stabilizes Env in a prefusion “closed” conformation that is preferentially targeted by several broadly neutralizing antibodies (bNAbs). A recent study showed that an analog of temsavir (BMS-377806) affects the cleavage and addition of complex glycans on Env. In this study, we investigated the impact of temsavir on the overall glycosylation, proteolytic cleavage, cell surface expression, and antigenicity of Env. We found that temsavir impacts Env glycosylation and processing at physiological concentrations. This significantly alters the capacity of several bNAbs to recognize Env present on virions and HIV-1-infected cells. Temsavir treatment also reduces the capacity of bNAbs to eliminate HIV-1-infected cells by antibody-dependent cellular cytotoxicity (ADCC). Consequently, the impact of temsavir on Env glycosylation and antigenicity should be considered for the development of new antibody-based approaches in temsavir-treated individuals.

## OBSERVATION

HIV-1 envelope glycoproteins (Env) mediate viral entry and are synthetized as a gp160 precursor, which is then trimerized and cleaved by host furin-like proteases (1, 2). This generates the mature Env composed of three gp120 exterior and three gp41 transmembrane subunits. Env is the only virus-specific antigen on the surface of the virus and infected cells, and as such, it is the target of neutralizing and nonneutralizing antibodies (nnAbs) (3). Over the years, significant developments have been made in the generation of HIV-1 entry inhibitors, such as maraviroc (CCR5 antagonist), enfuvirtide (gp41 fusion inhibitor), and ibalizumab (CD4 antagonist): however, none of them directly targets gp120 (4–6). In July 2020, a new small molecule attachment inhibitor, fostemsavir (BMS-663068 [Rukobia]), obtained FDA approval to treat HIV-1-infected individuals who had developed multidrug resistance (7).

Temsavir (BMS-626529), the active compound of fostemsavir, binds a conserved pocket under the β20-β21 loop of gp120 (8). The binding of this drug prevents the interaction with CD4 and stabilizes Env in a prefusion “closed” state 1 conformation, which is preferentially targeted by broadly neutralizing antibodies (bNAbs) (8–11). By stabilizing Env in this “closed” conformation, temsavir might be helpful in exposing Env in its untriggered native state to the immune system. However, an analog of temsavir, BMS-377806, was recently shown to decrease the cleavage of gp160 as well as the addition of complex glycans, two processes related to Env conformational flexibility (12). Since most bNAbs preferentially recognize conformation-dependent epitopes that are composed of or adjacent to glycans, we evaluated the impact of temsavir on overall Env glycosylation, its proteolytic cleavage, and the binding capacity of bNAbs to Env present on the surface of virions and infected primary CD4^+^ T cells. Since the combination of temsavir with certain bNAbs is being explored (13), we also evaluated whether temsavir treatment affected the capacity of bNAbs to eliminate infected cells by antibody-dependent cellular cytotoxicity (ADCC).

**FIG 1:**
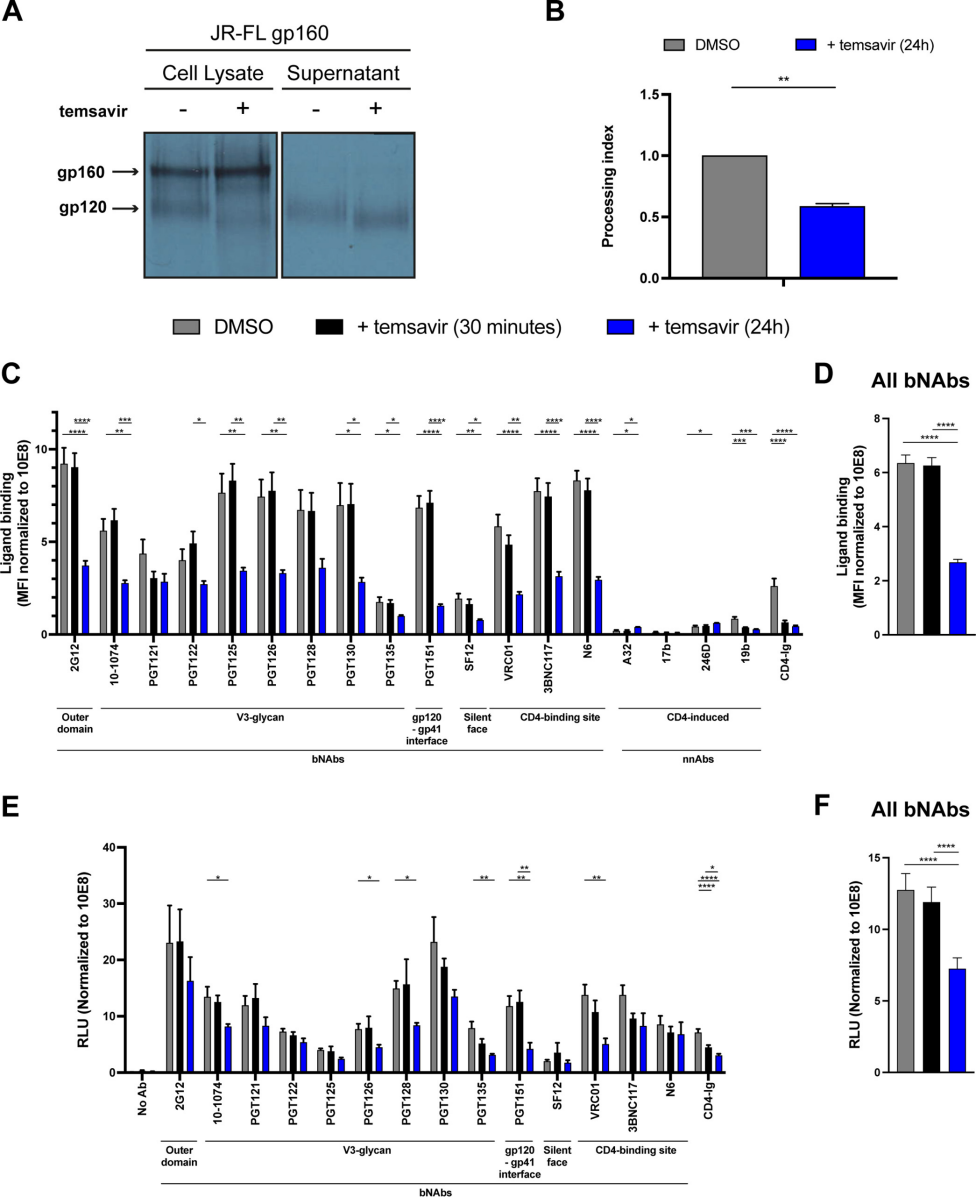
Temsavir alters Env glycosylation, cleavage, and bNAb binding. (A) HEK 293T cells were transfected with a plasmid expressing JR-FL Env and metabolically labeled for 24 h with [^35^S]methionine and [^35^S]cysteine in the presence of 10 μM temsavir or the equivalent volume of DMSO. Cell lysates and supernatants were immunoprecipitated with plasma from HIV-1-infected individuals. The precipitated proteins were loaded onto SDS-PAGE gels and analyzed by autoradiography. (B) Quantification of the impact of temsavir on Env processing on Env-expressing HEK 293T cells in cell lysates and supernatants. (C) HEK 293T cells were transfected with a plasmid expressing JR-FL Env together with a plasmid expressing the GF and treated with 10 μM temsavir for 24 h or the equivalent volume of DMSO. Cells were than stained for recognition of cell-surface Env by the indicated ligands in the presence (30 min) or absence of temsavir (10 μM). Shown are the mean fluorescence intensities (MFI) using the different ligands normalized to the signal obtained with the glycan-independent 10E8 MAb. MFI values were measured on the transfected (green fluorescent protein-positive [GFP^+^]) population. (D) The graph shown represents the compilation of normalized MFI for all bNAbs for each condition. (E) The capacity of the indicated ligands to capture viral particles bearing HIV-1_JR-FL_ Env was assessed by a virus capture assay. Temsavir was added for 24 h on virus-producing cells or only during the assay (30 min). Relative light units (RLU) obtained using a given ligand were normalized to the signal obtained with the 10E8 MAb. (F) The graph shown represents the compilation of normalized RLU for all bNAbs for each condition. Error bars indicate the mean ± standard error of the mean (SEM). The data shown are representative of results from at least three independent experiments. Statistical significance was tested using (B) a Mann-Whitney test or (C to F) one-way analysis of variance (ANOVA) (*, *P* < 0.05; **, *P* < 0.01; ***, *P* < 0.001; ****, *P* < 0.0001).

Because BMS-377806 limits Env conformational flexibility (12), we first investigated the impact of temsavir on overall Env processing and glycosylation. To do this, HEK 293T cells were transfected with a plasmid encoding the gp160 of the primary tier 2 JR-FL isolate. This Env was selected since it has been extensively characterized structurally (14), used for ADCC responses (15, 16), and was previously used to measure the impact of short-term temsavir treatment on the conformational landscape of incorporated Env (9, 10). Transfected cells were radioactively labeled, followed by immunoprecipitation of whole-cell lysates and supernatants with HIV^+^ plasma, as previously reported (17). We observed an impact of temsavir on gp120 glycosylation, as illustrated by the presence of a lower-molecular-weight band of gp120 in the cell lysate and the supernatant from treated cells (Fig. 1A). This phenotype was dose dependent and observed at biologically relevant doses (see Fig. S1A in the supplemental material) since its effect was visualized at 100 nM, which is below the concentration achieved in treated individuals (18). As previously observed with BMS-377806, temsavir treatment also significantly (*P* < 0.01) reduced Env processing (12) (Fig. 1A and B; Fig. S1A).

10.1128/mbio.00577-22.2 FIG S1 Temsavir’s effect on Env glycosylation and ligand binding at different concentrations. (A) HEK 293T cells were transfected with a plasmid expressing JR-FL Env and metabolically labeled for 24 h with [^35^S]methionine and [^35^S]cysteine in the presence of increasing concentrations of temsavir (0.1, 0.5, 1, 5, and 10 μM) or the equivalent volume of DMSO. Cell lysates and supernatants were immunoprecipitated with plasma from HIV-1-infected individuals. The precipitated proteins were loaded onto SDS-PAGE gels and analyzed by autoradiography (B) HEK 293T cells were transfected with a plasmid expressing JR-FL Env together with a plasmid expressing the green fluorescence protein (GFP) and treated with increasing concentrations of temsavir (0.001, 0.01, 0.1, 1, and 10 μM) for 24 h or with the equivalent volume of DMSO. Cells were then stained for recognition of cell surface Env by 2G12 and PGT151. Shown are the mean fluorescence intensities (MFI) using the different ligands normalized to the signal obtained with the glycan-independent 10E8 MAb. MFI values were measured on the transfected (GFP^+^) population. Download FIG S1, PDF file, 0.5 MB. Copyright © 2022 Boutin et al. 2022 Boutin et al. https://creativecommons.org/licenses/by/4.0/ This content is distributed under the terms of the Creative Commons Attribution 4.0 International license.

Having corroborated that treatment of Env-expressing cells with temsavir decreases Env glycosylation and cleavage, we then evaluated whether it affects its recognition by a panel of bNAbs and nonneutralizing antibodies. Our panel comprises the gp120 outer domain glycan-dependent 2G12 antibody (Ab), V3 glycan (10-1074, PGT121, PGT122, PGT125, PGT126, PGT128, PGT130, and PGT135), gp120-gp41 interface (PGT151), silent face (SF12), CD4-binding site (VRC01, 3BNC117, and N6), as well as CD4-induced (CD4i) antibodies (A32, 17b, 246D, and 19b). The CD4-Ig protein was used as a readout for CD4 binding. HEK 293T cells were transfected with a plasmid expressing the full-length HIV-1 isolate JR-FL Env wild type (WT) and subsequently treated with temsavir for 24 h. To differentiate the impact of temsavir on Env glycosylation/cleavage from its state 1 stabilizing effect, temsavir was also added only during the 30-min incubation period with monoclonal antibodies (MAbs). Since no major effects were observed with the anti-gp41 MPER 10E8 bNAb upon short or long treatment (see Fig. S2 in the supplemental material), we used this antibody to normalize cell surface Env expression. We observed no major changes in the recognition of Env by our panel of bNAbs when temsavir was added at the same time as the antibodies. Remarkably, long temsavir treatment (24 h) significantly decreased recognition by several bNAbs targeting the outer domain, the V3-glycans, the gp120-gp41 interface, or the CD4-binding site (*P* < 0.0001) (Fig. 1C and D), thus, suggesting that modulation of Env glycosylation/cleavage by temsavir significantly affects Env antigenicity. The intrinsic contribution of each of these mechanisms (i.e., impaired Env cleavage and/or glycosylation changes) to the overall decrease in bNAb recognition remains unknown. Importantly, this effect was dose dependent and observed with concentrations as low as 10 nM (Fig. S1B). While temsavir also reduced CD4-Ig binding, this phenotype could be linked to its capacity to compete with CD4 and the treatment’s effect on glycosylation (8), especially since CD4-Ig binding was also reduced upon short-term temsavir treatment. As previously reported, nonneutralizing epitopes were poorly exposed at the surface of Env-expressing cells (19). Env proteolytic cleavage decreases its flexibility and exposure of epitopes recognized by nnAbs (15, 20, 21). Accordingly, uncleaved Envs are readily recognized by CD4i nnAbs (20). Despite its effect on Env cleavage, temsavir treatment did not significantly increase Env recognition by CD4i nnAbs (Fig. 1C). This is likely due to its capacity to stabilize Env in its “closed” state 1 conformation (10). Importantly, the impact of temsavir on Env antigenicity was specific since no effect was observed with the temsavir-resistant Env S375W mutant (see Fig. S3 in the supplemental material) (22).

10.1128/mbio.00577-22.3 FIG S2 Normalization of cell surface Env expression by anti-gp41 MPER 10E8 bNAb. HEK 293T cells were transfected with a plasmid expressing JR-FL Env together with a plasmid expressing the GFP and treated with 10 μM temsavir for 24 h or the equivalent volume of DMSO. Cells were than stained for recognition of cell surface Env by the indicated ligands in the presence (30 min) or absence of temsavir (10 μM). Shown are the mean fluorescence intensities (MFI) using the glycan-independent 10E8 MAb. MFI values were measured on the transfected (GFP^+^) population. Statistical significance was measured using ordinary one-way ANOVA. The data shown are representative of results from 14 experiments. Download FIG S2, PDF file, 0.3 MB. Copyright © 2022 Boutin et al. 2022 Boutin et al. https://creativecommons.org/licenses/by/4.0/ This content is distributed under the terms of the Creative Commons Attribution 4.0 International license.

10.1128/mbio.00577-22.4 FIG S3 Impact of temsavir treatment on the recognition of the Env S375W mutant by bNAbs. HEK 293T cells were transfected with a plasmid expressing JR-FL Env WT together with a plasmid expressing the GFP or the temsavir-resistant S375W mutant. Cells were treated with 10 μM temsavir for 24 h or the equivalent volume of DMSO. Cells were than stained for recognition of cell surface Env by the indicated ligands in the presence or absence of temsavir (10 μM). Shown are the mean fluorescence intensities (MFI) using the different ligands normalized to the signal obtained with the glycan-independent 10E8 MAb. MFI values were measured on the transfected (GFP^+^) population. Error bars indicate the mean ± SEM. The data shown are representative of results from five experiments. Statistical significance was tested using ordinary one-way ANOVA (*, *P* < 0.05; **, *P* < 0.01; ***, *P* < 0.001; ****, *P* < 0.0001). Download FIG S3, PDF file, 0.4 MB. Copyright © 2022 Boutin et al. 2022 Boutin et al. https://creativecommons.org/licenses/by/4.0/ This content is distributed under the terms of the Creative Commons Attribution 4.0 International license.

To evaluate whether temsavir treatment also affected the capacity of bNAbs to bind Env presented at the surface of viral particles, we used a previously described virus capture assay (VCA) (23). Viral particles expressing the JR-FL Env produced in the presence or absence of temsavir were added to plates coated with bNAbs or CD4-Ig, and viral capture was measured as previously described (23). Similar to the observations with Env-expressing cells, temsavir treatment of virus-producing cells significantly decreased Env recognition by multiple bNAbs and ligands, including 10-1074 (*P* < 0.05), PGT126 (*P* < 0.05), PGT128 (*P* < 0.05), PGT151 (*P* < 0.01), VRC01 (*P* < 0.01), and CD4-Ig (*P* < 0.0001) (Fig. 1E). Taking all tested bNabs together, a significant reduction of binding was observed upon 24 h of temsavir treatment (*P* < 0.0001) (Fig. 1F). In contrast, short-term (30 min) temsavir treatment did not significantly reduce the capacity of bNabs to capture viral particles bearing JR-FL Env. This result suggests that the impact of temsavir on Env glycosylation and/or cleavage, rather than its direct impact on Env conformation, decreases the capacity of tested anti-Env ligands to bind viral particles.

**FIG 2:**
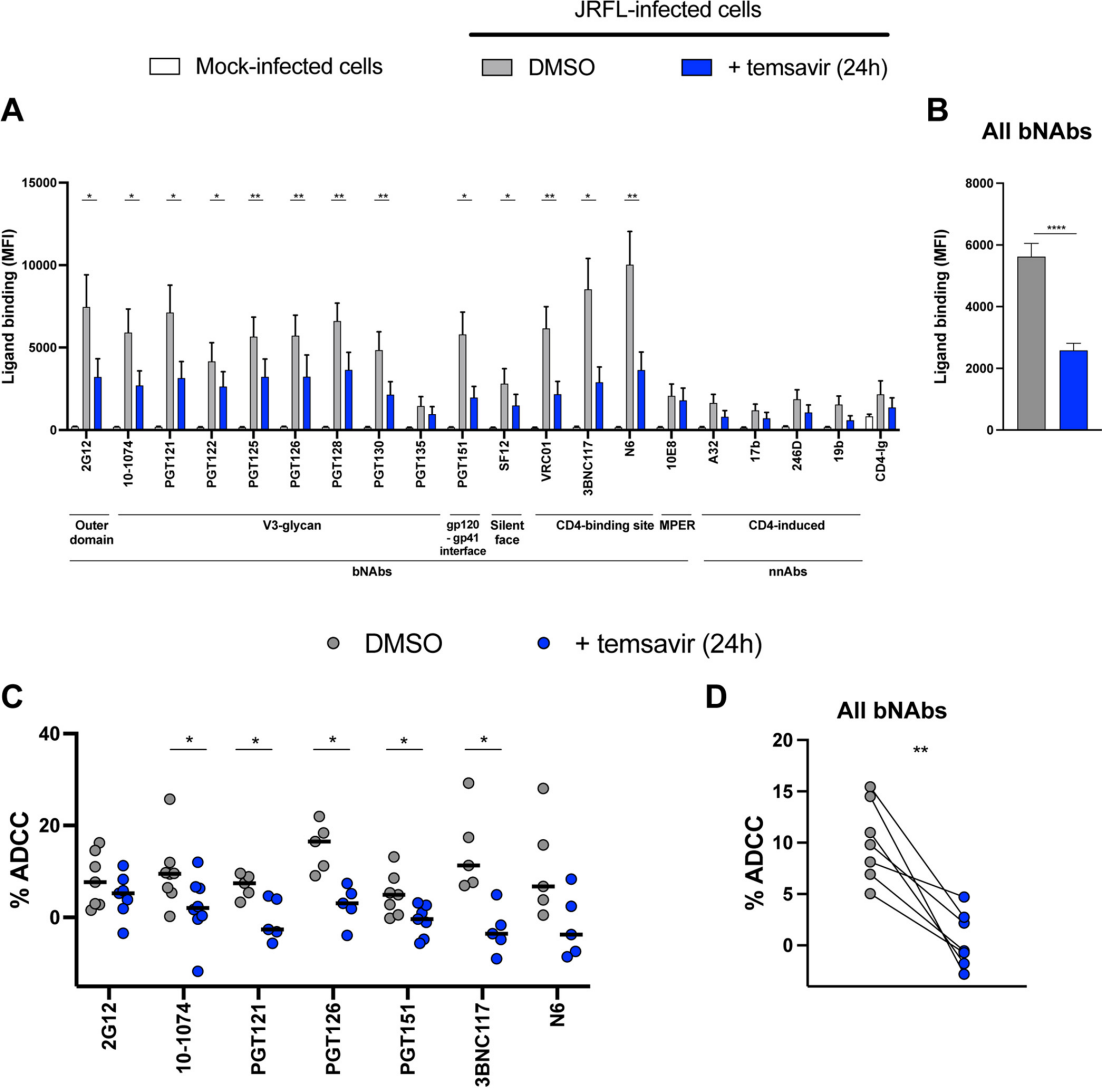
Impact of temsavir treatment on the recognition and elimination of infected primary CD4^+^ T cells by ADCC. (A) Primary CD4^+^ T cells infected with HIV-1_JR-FL_ virus were treated with 10 μM temsavir for 24 h or the equivalent volume of DMSO and were stained for recognition of cell surface Env by the indicated ligands. Mock-infected cells were used as a control for anti-Env ligand specificity. Shown are the mean fluorescence intensities (MFI) measured on the infected (p24^+^) population of the average of 3 different donors. (B) The graph shown represents the compilation of MFI for all bNAbs for each condition. (C) Primary CD4^+^ T cells infected with HIV-1_JR-FL_ virus were used as target cells and autologous peripheral blood mononuclear cells (PBMCs) as effector cells in a FACS-based ADCC assay. The graphs shown represent the percentages of ADCC mediated by 2G12, 10-1074, PGT121, PGT126, PGT151, 3BNC117, and N6 with target cells treated for 24 h with 10 μM temsavir or the equivalent volume of DMSO. (D) The graph shown represents the mean percentage of ADCC obtain for each tested antibody with DMSO and temsavir treatment. Ligand binding and ADCC responses were obtained in at least 5 independent experiments using cells from 6 different donors. Error bars indicate means ± SEM. Statistical significance was tested using a paired *t* test (*, *P* < 0.05; **, *P* < 0.01; ***, *P* < 0.001; ****, *P* < 0.0001).

To evaluate the impact of temsavir treatment on the capacity of bNAbs to recognize HIV-1-infected cells, we used HIV-1_JR-FL_-infected primary CD4^+^ T cells subjected to temsavir or dimethyl sulfoxide (DMSO) treatment for 24 h prior to measuring MAb binding. Consistent with our observations obtained using Env-expressing HEK 293T cells, productively infected cells (p24^+^) were significantly less recognized by several bNAbs upon temsavir treatment (*P* < 0.0001) (Fig. 2A and B).

With this phenotype, we next evaluated whether the capacity of bNAbs to eliminate infected cells by ADCC was also affected. After a 24 h treatment, ADCC mediated by the bNAbs 2G12, 10-1074, PGT121, PGT126, PGT151, 3BNC117, and N6 was measured using a fluorescence-activated cell sorter (FACS)-based assay that measures the elimination of productively infected cells (24). In agreement with decreased recognition, temsavir-treated cells were significantly more resistant to ADCC responses mediated by the majority of these bNAbs (*P* < 0.01) (Fig. 2C and D).

In summary, we have demonstrated that temsavir treatment of Env-producing cells alters the overall antigenicity of Env present at the surface of virions and infected cells. This information is important for the development of immunotherapies aimed at decreasing the size of the viral reservoir in temsavir-treated individuals.

Analyses of Env conformation and ADCC responses were performed as described in detail in Text S1 in the supplemental material.

10.1128/mbio.00577-22.1 TEXT S1 Supplemental materials and methods. Download Text S1, DOCX file, 0.03 MB. Copyright © 2022 Boutin et al. 2022 Boutin et al. https://creativecommons.org/licenses/by/4.0/ This content is distributed under the terms of the Creative Commons Attribution 4.0 International license.
