# Supplementary figures and images for: Temsavir Treatment of HIV-1-Infected Cells Decreases Envelope Glycoprotein Recognition by Broadly Neutralizing Antibodies
Source: mBio. 2022 Apr 27;13(3):e00577-22. doi: 10.1128/mbio.00577-22 (PMC9239219; doi:10.1128/mbio.00577-22)

**A**

JR-FL gp160

Cell Lysate

Supernatant

temsavir ( $\mu\text{M}$ )

0 0.1 0.5 1 5 10

0 0.1 0.5 1 5 10

gp160 →  
gp120 →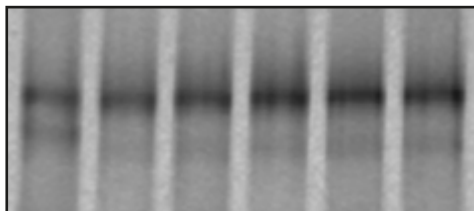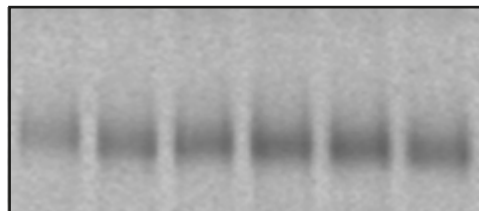**B**

2G12

PGT151

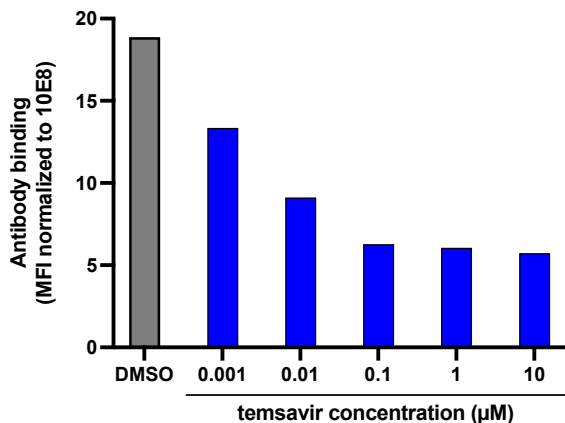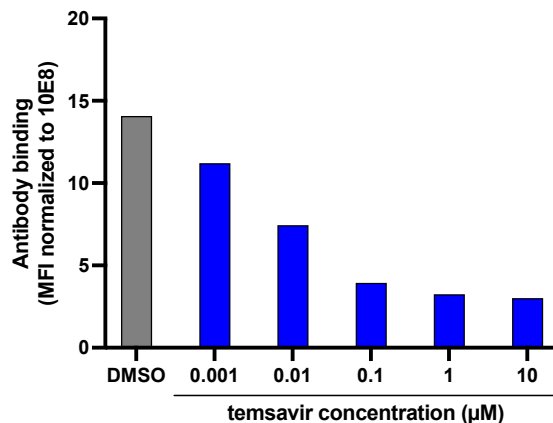

DMSO

+ temsavir (24h)

Supplement: FIG S1 [file mbio.00577-22-s0002.pdf]

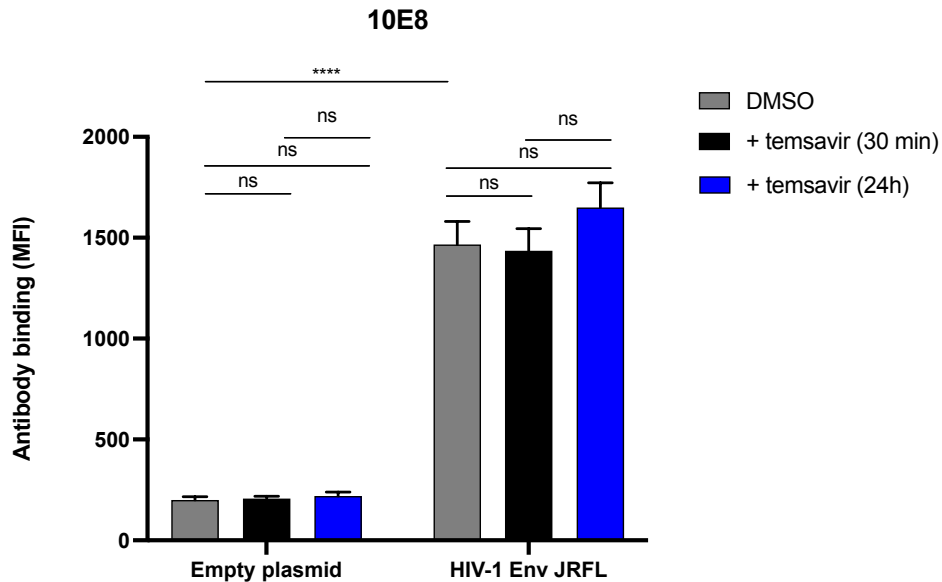

Supplement: FIG S2 [file mbio.00577-22-s0003.pdf]

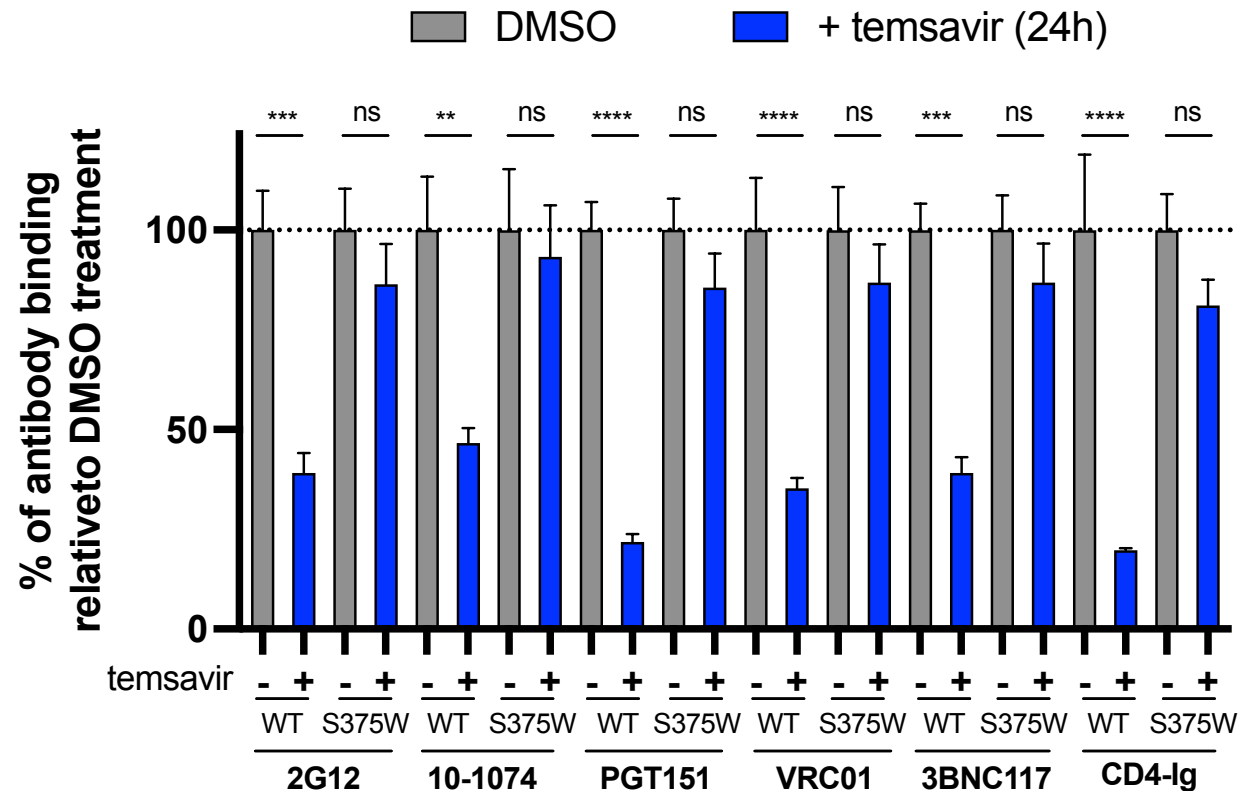

Supplement: FIG S3 [file mbio.00577-22-s0004.pdf]
