# Supplementary material for: Temsavir Treatment of HIV-1-Infected Cells Decreases Envelope Glycoprotein Recognition by Broadly Neutralizing Antibodies
Source: mBio. 2022 Apr 27;13(3):e00577-22. doi: 10.1128/mbio.00577-22 (PMC9239219; doi:10.1128/mbio.00577-22)
Supplement: TEXT S1 [file mbio.00577-22-s0001.docx]

**SUPPLEMENTAL MATERIAL**

Supplemental material is available online.

**Ethics Statement**

Written informed consent was obtained from all study participants and research adhered to the ethical guidelines of CRCHUM and was reviewed and approved by the CRCHUM institutional review board (ethics committee, approval number CE 16.164 - CA). Research adhered to the standards indicated by the Declaration of Helsinki. All participants were adult and provided informed written consent prior to enrolment in accordance with Institutional Review Board approval.

**Cell lines and primary cells.**

HEK 293T human embryonic kidney cells (obtained from ATCC) were maintained at 37°C under 5% CO_2_ in Dulbecco's modified Eagle's medium (DMEM; Wisent) containing 5% fetal bovine serum (FBS; VWR) and 100 μg/mL of penicillin–streptomycin (Wisent). Primary human PBMCs and CD4+ T cells were isolated, activated and cultured as previously described (1). Briefly, PBMCs were obtained by leukapheresis from seven HIV-negative individuals (five males and two females). CD4+ T cells were purified from rested PBMCs by negative selection kit (EasySep human CD4+ T cell enrichment kit; STEMCELL Technologies) and were activated with phytohemagglutinin-L (10µg/mL) for 48 hours and then maintained in RPMI 1640 complete medium supplemented with rIL-2 (100 U/mL).

**Plasmids.**

The sequence of full-length clade B JR-FL Env was codon optimized (GenScript) and cloned into expression plasmid pcDNA3.1(-) (Invitrogen) (2). The S375W mutation was introduced in the JR-FL Env expressor using the QuikChange II XL site-directed mutagenesis protocol (Agilent Technologies) (3). The presence of the desired mutations was determined by automated DNA sequencing. The HIV-1_JR-FL_ IMC was previously described (4). Vesicular stomatitis virus G (VSV-G)-encoding plasmid was also previously reported (5). The reporter proviral vector pNL4.3 Luc R-E- was obtained through the NIH AIDS Reagent Program.

**Antibodies.**

The following Abs were used as primary antibodies for cell-surface Env staining: 10e8, 2G12, VRC01, N6, CD4-Ig, A32, 17b and 19b (NIH AIDS Reagent Program), 10-1074, 3BNC117, SF12 (kindly provided by Michel Nussenzweig), 246D (kindly provided by Susan Zolla-Pazner), PGT121, PGT122, PGT125, PGT126, PGT128, PGT130, PGT135, PGT151 (IAVI) and the soluble CD4-immunoglobulin fusion protein (CD4-Ig) (NIH AIDS Reagent Program). Goat anti-human IgG (H+L) antibodies pre-coupled to Alexa Fluor 647 (Invitrogen) were used as secondary antibodies in flow cytometry experiments.

**Small molecules.**

The HIV-1 attachment inhibitor temsavir (BMS-626529) was purchased from APExBIO. The compounds were dissolved in dimethyl sulfoxide (DMSO) at a stock concentration of 10 mM and diluted to 10 µM in phosphate-buffered saline (PBS) for cell-surface staining and virus capture assay or RPMI 1640 complete medium for ADCC assays or for 24h treatment.

**Radioactive labeling and immunoprecipitation of envelope glycoproteins.**

HEK 293T cells (5 × 10^5^) were transfected by the calcium phosphate method with the JR-FL Env expressor. One day after transfection, cells were metabolically labeled for 16-24 h with 100 μCi/mL of [^35^S] methionine-cysteine ([^35^S] Protein Labeling Mix; PerkinElmer) in DMEM lacking methionine and cysteine and supplemented with 10% dialyzed fetal bovine serum and 1% GlutaMAX™ supplement (Thermo Fisher Scientific). Cells were subsequently lysed in RIPA buffer (140 mM NaCl, 8 mM Na_2_HPO_4_, 2 mM NaH_2_PO_4_, 1% IGEPAL® CA-630 (Sigma-Aldrich), 0.05% sodium dodecyl sulfate (SDS), 1.2 mM sodium deoxycholate). Precipitation of radiolabeled envelope glycoproteins from the whole-cell lysates or found in the supernatant was performed with a pool of sera from HIV-1-infected individuals in the presence of 45 μL of 10% Protein A-Sepharose beads (Cytiva) at 4 °C. The precipitated proteins were loaded onto SDS-PAGE gels and analyzed by autoradiography and densitometry to calculate their processing indices. The processing index is a measure of the conversion of the temsavir-treated gp160 Env precursor to mature gp120, relative to mock-treated Env trimers and takes into account the total amount of Env-related bands present in the cell lysate and supernatant. The processing index is calculated with the following formula: processing index = ([total gp120]treated × [gp160]mock-treated)/([gp160]treated × [total gp120]mock-treated). Total refers to the gp120 in the cell lysate and supernatant.

**Transfection.**

HEK 293T cells (3 × 10^5^) were transfected with 10 μg of a plasmid expressing JR-FL WT gp160, JRFL S375W gp160 (or pcDNA3.1 as negative control) and 2.5 μg of a green fluorescent protein (GFP) expressor (pIRES2‐GFP; Clontech) using the standard calcium phosphate method. The next day, media were changed for fresh media containing 10µM of temsavir (temsavir 24h) or the equivalent volume of DMSO (conditions DMSO and temsavir 30 min)

**Virus production and infections.**

To obtain the same level of infection among the different experiments, vesicular stomatitis virus G (VSV-G)-pseudotyped HIV-1_JR-FL_ viruses were produced and titrated as previously described (6). HIV-1_JR-FL_ viruses were used to infect activated primary CD4^+^ T cells from healthy donors by spin infection at 800 × *g* for 1h in 96-well plates at room temperature.

**Virus capture assay.**

The assay was modified from a previous published method (7). Briefly, pseudoviral particles were produced by transfecting 2×10^6^ HEK 293T cells with pNL4.3 Luc R-E- (3.5 μg), plasmids encoding for JR-FL WT gp160 or JR-FL S375W gp160 (3.5 μg) and VSV-G (1μg) using the standard calcium phosphate protocol. Twenty-four hours later, media of the transfected cells was changed with full DMEM medium containing 10µM temsavir or the equivalent volume of DMSO. Virion-containing supernatants were collected after 24h treatment and cell debris were removed through centrifugation (486 × g for 10 min). Half of the supernatant treated with DMSO was incubated with 10µM temsavir for 30 minutes before adding to the ELISA plate. To immobilize antibodies on ELISA plates, white MaxiSorp ELISA plates (Thermo Fisher Scientific) were incubated with 5 μg/mL of antibodies in 100 μL PBS overnight at 4℃. Unbound antibodies were removed by washing the plates twice with PBS. Plates were subsequently blocked with 3% bovine serum albumin (BSA) in PBS for 1h at room temperature. After two washes with PBS, 200 μL of virion-containing supernatant were added per well. After 4 to 6 hours incubation, supernatants were removed, and the wells were washed with PBS 3 times. Viral capture by any given antibody was visualized by adding 1 ×10^4^ HIV-1 resistant 293T cells in full DMEM medium per well. Forty-eight hours post-infection, cells were lysed by the addition of 30 μL of passive lysis buffer (Promega) and one freeze-thaw cycle. An LB942 TriStar luminometer (Berthold Technologies) was used to measure the luciferase activity of each well after the addition of 100 μL of luciferin buffer (15 mM MgSO_4_, 15 mM KH_2_PO_4_ [pH 7.8], 1 mM ATP, and 1mM dithiothreitol) and 50 μL of 1 mM D-luciferin potassium salt (Prolume).

**Flow cytometry analysis of cell-surface staining**

For cell surface staining, HEK 293T or primary CD4^+^ T cells were incubated for 30 min at 37°C 48h post-transfection/infection with 5 μg/mL of anti-Env monoclonal antibodies in PBS. Cells were then washed twice with PBS and stained with 2 μg/mL goat anti-human IgG Alexa Fluor 647 secondary antibodies for 20 min in PBS. After two more PBS washing, cells were fixed in a 2% PBS-formaldehyde solution. For the detection of intracellular p24 protein, infected primary CD4+ T cells were permeabilized using the Cytofix/Cytoperm Fixation/Permeabilization Kit (BD Biosciences) and stained intracellularly using the PE-conjugated mouse anti-p24 mAb (clone KC57; Beckman Coulter; 1:100 final concentration). The percentage of infected cells (p24^+^ cells) was determined by gating the living cell population on the basis of viability dye staining (AquaVivid, Thermo Fisher Scientific). Samples were analyzed on an LSRII cytometer (BD Biosciences), and data analysis was performed using FlowJo v10.5.3 (Tree Star).

**FACS-based ADCC assay**

Measurement of ADCC using the FACS-based assay was performed at 48h post- infection as previously described (1, 8). Briefly, HIV-1-infected primary CD4+ T cells were stained with AquaVivid viability dye and cell proliferation dye (eFluor670; eBioscience) and used as target cells. Autologous PBMC effector cells, stained with another cellular marker (cell proliferation dye eFluor450; eBioscience), were added at an effector: target ratio of 10:1 in 96-well V-bottom plates (Corning). For evaluation of antibody-dependent cellular cytotoxicity (ADCC), 5 µg/mL of anti-Env mAbs were added to appropriate wells and cells were incubated for 5 min at room temperature. The plates were subsequently centrifuged for 1 min at 300 × g, and incubated at 37°C, 5% CO_2_ for 5 h before being fixed in a 2% PBS-formaldehyde solution. Cells were than permeabilized using the Cytofix/Cytoperm Fixation/Permeabilization Kit (BD Biosciences) and stained intracellularly using the PE-conjugated mouse anti-p24 mAb (clone KC57; Beckman Coulter; 1:100 final concentration). Samples were acquired on an LSRII cytometer (BD Biosciences) and data analysis was performed using FlowJo v10.5.3 (Tree Star). The percentage of ADCC was calculated with the following formula: (% of p24+ cells in Targets plus Effectors) − (% of p24+ cells in Targets plus Effectors plus antibody) / (% of p24+ cells in Targets) by gating on infected lived target cells.

**Statistical analyses**

Statistics were analyzed using GraphPad Prism version 9.1.0 (GraphPad). Every dataset was tested for statistical normality and this information was used to apply the appropriate (parametric or nonparametric) statistical test. P values < 0.05 were considered significant; significance values are indicated as ∗ p < 0.05, ∗∗ p < 0.01, ∗∗∗ p < 0.001, ∗∗∗∗ p < 0.0001. Corrections for multiple comparisons were performed with the Holm-Sidak post-test.

**References**

1. Veillette M, Désormeaux A, Medjahed H, Gharsallah N-E, Coutu M, Baalwa J, Guan Y, Lewis G, Ferrari G, Hahn BH. 2014. Interaction with cellular CD4 exposes HIV-1 envelope epitopes targeted by antibody-dependent cell-mediated cytotoxicity. Journal of virology 88:2633-2644.

2. Mao Y, Wang L, Gu C, Herschhorn A, Xiang S-H, Haim H, Yang X, Sodroski J. 2012. Subunit organization of the membrane-bound HIV-1 envelope glycoprotein trimer. Nature structural & molecular biology 19:893-899.

3. Prévost J, Tolbert WD, Medjahed H, Sherburn RT, Madani N, Zoubchenok D, Gendron-Lepage G, Gaffney AE, Grenier MC, Kirk S, Vergara N, Han C, Mann BT, Chénine AL, Ahmed A, Chaiken I, Kirchhoff F, Hahn BH, Haim H, Abrams CF, Smith AB, 3rd, Sodroski J, Pazgier M, Finzi A. 2020. The HIV-1 Env gp120 Inner Domain Shapes the Phe43 Cavity and the CD4 Binding Site. mBio 11.

4. O'Brien WA, Koyanagi Y, Namazie A, Zhao JQ, Diagne A, Idler K, Zack JA, Chen IS. 1990. HIV-1 tropism for mononuclear phagocytes can be determined by regions of gp120 outside the CD4-binding domain. Nature 348:69-73.

5. Emi N, Friedmann T, Yee JK. 1991. Pseudotype formation of murine leukemia virus with the G protein of vesicular stomatitis virus. J Virol 65:1202-7.

6. Veillette M, Coutu M, Richard J, Batraville L-A, Dagher O, Bernard N, Tremblay C, Kaufmann DE, Roger M, Finzi A. 2015. The HIV-1 gp120 CD4-bound conformation is preferentially targeted by antibody-dependent cellular cytotoxicity-mediating antibodies in sera from HIV-1-infected individuals. Journal of virology 89:545-551.

7. Ding S, Gasser R, Gendron-Lepage G, Medjahed H, Tolbert WD, Sodroski J, Pazgier M, Finzi A. 2019. CD4 Incorporation into HIV-1 Viral Particles Exposes Envelope Epitopes Recognized by CD4-Induced Antibodies. J Virol 93.

8. Richard J, Veillette M, Brassard N, Iyer SS, Roger M, Martin L, Pazgier M, Schön A, Freire E, Routy JP, Smith AB, 3rd, Park J, Jones DM, Courter JR, Melillo BN, Kaufmann DE, Hahn BH, Permar SR, Haynes BF, Madani N, Sodroski JG, Finzi A. 2015. CD4 mimetics sensitize HIV-1-infected cells to ADCC. Proc Natl Acad Sci U S A 112:E2687-94.
